# Graves’ disease presenting as bi - ventricular heart failure with severe pulmonary hypertension and pre-eclampsia in pregnancy – a case report and review of the literature

**DOI:** 10.1186/1756-0500-7-814

**Published:** 2014-11-18

**Authors:** Khandker Mohammad Nurus Sabah, Abdul Wadud Chowdhury, Mohammad Shahidul Islam, Fathima Aaysha Cader, Shamima Kawser, Md Imam Hosen, Mohammed Abaye Deen Saleh, Md Shariful Alam, Mohammad Monjurul Kader Chowdhury, Humayara Tabassum

**Affiliations:** Department of Cardiology, Dhaka Medical College Hospital, Dhaka, Bangladesh; Department of Microbiology, Dr. Sirajul Islam Medical College, Dhaka, Bangladesh

**Keywords:** Graves’ disease, Hypertension, Bi- Ventricular Heart Failure, Pulmonary Hypertension, Pre-eclampsia, Pregnancy

## Abstract

**Background:**

Graves’ disease, a well-known cause of hyperthyroidism, is an autoimmune disease with multi-system involvement. More prevalent among young women, it appears as an uncommon cardiovascular complication during pregnancy, posing a diagnostic challenge, largely owing to difficulty in detecting the complication, as a result of a low index of suspicion of Graves’ disease presenting during pregnancy. Globally, cardiovascular disease is an important factor for pregnancy-related morbidity and mortality. Here, we report a case of Graves’ disease detected for the first time in pregnancy, in a patient presenting with bi- ventricular heart failure, severe pulmonary hypertension and pre- eclampsia. Emphasis is placed on the spectrum of clinical presentations of Graves’ disease, and the importance of considering this thyroid disorder as a possible aetiological factor for such a presentation in pregnancy.

**Case presentation:**

A 30-year-old Bangladeshi-Bengali woman, in her 28th week of pregnancy presented with severe systemic hypertension, bi-ventricular heart failure and severe pulmonary hypertension with a moderately enlarged thyroid gland. She improved following the administration of high dose intravenous diuretics, and delivered a premature female baby of low birth weight per vaginally, twenty four hours later. Pre-eclampsia was diagnosed on the basis of hypertension first detected in the third trimester, 3+ oedema and mild proteinuria. Electrocardiography revealed sinus tachycardia with incomplete right bundle branch block and echocardiography showed severe pulmonary hypertension with an estimated pulmonary arterial systolic pressure of 73 mm Hg, septal and anterior wall hypokinesia with an ejection fraction of 51%, grade I mitral and tricuspid regurgitation. Thyroid function tests revealed a biochemically hyperthyroid state and positive anti- thyroid peroxidase antibodies was found. ^99m^Technetium pertechnetate thyroid scans demonstrated diffuse toxic goiter as evidenced by an enlarged thyroid gland with intense radiotracer concentration all over the gland. The clinical and biochemical findings confirmed the diagnosis of Graves’ disease.

**Conclusions:**

Graves’ disease is an uncommon cause of bi-ventricular heart failure and severe pulmonary hypertension in pregnancy, and a high index of clinical suspicion is paramount to its effective diagnosis and treatment.

## Background

### Case report

An autoimmune disease with female predilection, Graves’ disease (GD) is the most common cause of hyperthyroidism, and is associated with multisystem involvement. Chiefly characterized by a diffuse goitre and features of thyrotoxicosis, it may also be accompanied by an infiltrative orbitopathy, ophthalmopathy and occasionally infiltrative dermopathy. Due to the autoimmune nature of GD, and given that pregnancy is a state of immunosuppression, thyrotoxic symptoms generally show a regression as the duration of pregnancy progresses. This can be explained by the diminished functions of both T-cells and B-cells under the influence of local placental factors and regulatory T cells. Globally, cardiovascular disease is an important factor for pregnancy-related morbidity and mortality, and complicates 1- 4% of all pregnancies [1, 2]. In addition to maternal mortality, cardiovascular diseases are responsible for approximately 30% of all deaths globally [3, 4]. GD is not a common presentation of cardiovascular complication in pregnancy. We report here a case of bi- ventricular heart failure, severe pulmonary and systemic hypertension and pre-eclampsia in pregnancy due to GD.

## Case presentation

**Figure 1:**
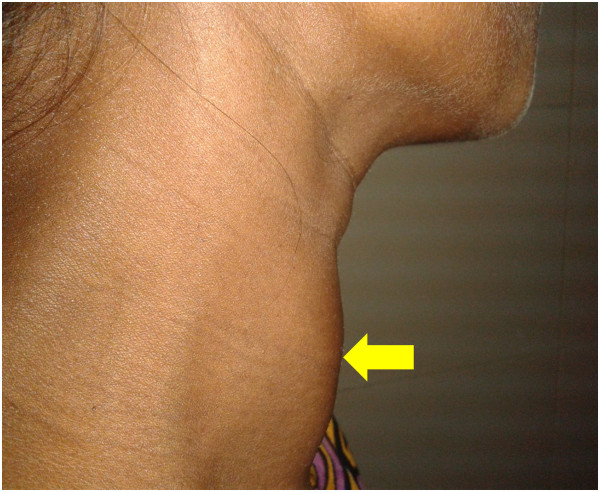
**Diffusely enlarged thyroid gland (indicated by arrow) in a 30-year-old female.**

This 30-year-old Bangladeshi-Bengali female in her 28th week of pregnancy was admitted to Dhaka Medical College Hospital with shortness of breath and raised blood pressure (BP - 160/100 mm of Hg). She had no prior history of hypertension. The following day she was transferred to cardiology owing to increased dyspnoea and altered level of consciousness. On initial assessment she was cyanosed and tachypnoeic with a respiratory rate of 50 breaths per min and blood pressure was raised at 180/100 mm Hg; there was significant bi-pedal oedema and bilateral pulmonary crackles on lung ausculation. She improved with initial treatment consisting of high doses intravenous diuretics and delivered a premature female baby of low birth weight (weight- 1.5 kg) per vaginally, twenty four hours later. Upon further evaluation, a moderately enlarged thyroid gland was detected. The goitre was diffuse, non-tender and mobile, with no features of compression, thrills or bruits (Figure 1). She had tachycardia (pulse132/min) and a regular, high volume pulse. However, there were no postural tremors, lid retraction, exophthalmos or other features of thyroid eye disease. Pulse pressure was wide (80 mm Hg). She had features of bi-ventricular heart failure with pulmonary hypertension, as evidenced by dependent oedema, raised jugular venous pressure, left ventricular gallop, bilateral basal lung crackles, and prominent pulmonary component of second heart sound on palpation and auscultation, with left parasternal heave; liver was enlarged and tender. Haemogram, serum electrolytes, serum creatinine and random blood sugar were within normal limit. Urine routine microscopy showed mild proteinuria with pus cells 4-5/HPF done before delivery. Electrocardiography showed sinus tachycardia with incomplete right bundle branch block and echocardiography revealed severe pulmonary hypertension with an estimated pulmonary arterial systolic pressure of 73 mm Hg, mild pericardial effusion (7 mm posteriorly), grade I mitral regurgitation (MR) & tricuspid regurgitation (TR), septal and anterior wall hypokinesia and fair left ventricular systolic function with an ejection fraction of 51%. Thyroid function tests revealed biochemically hyperthyroid state (Free T4- 4.26 ng/dl; Normal 0.71- 1.85 ng/dl and thyroid stimulation hormone [TSH] <0.015 uIU/ml; Normal 0.3 – 0.5 mIU/l). She had positive anti thyroid peroxidase antibodies (2107 μ/ml; Normal ≤15 μ/ml) and negative TSH receptor antibody. ^99m^Technetium pertechnetate thyroid scans confirmed a diffuse toxic goitre as evidenced by enlarged thyroid gland with intense radiotracer concentration all over the gland. Thus the clinical, biochemical and radiological features were consistent with Graves’ thyrotoxicosis. Pre-eclampsia was diagnosed on the basis of hypertension first detected in the third trimester, significant oedema and mild proteinuria. She responded to intravenous diuretics. Her thyroid hyperactivity was controlled with carbimazole 45 mg/day and propranolol 40 mg/day in divided doses, and blood pressure control following delivery was achieved with losartan potassium 50 mg/day. Her child also made a healthy recovery and they are both on regular follow up with the Cardiologist, Endocrinologist and Paediatrician.

### Discussion

Graves’ disease, named after Robert J. Graves was first described in 1825 by Dr. Caleb Hillier Parry [5, 6]. Known in Europe as von Basedow’s disease [7], it is an autoimmune disease that may occur at any age, with a peak incidence in the 40- to 60-year age group and female to male ratio of 5–10:1 [8].

In this case, the young age of onset of disease (at 30 years) and high levels of anti-thyroid peroxidase antibodies indicate the genetic and epigenetic factors involved in the pathogenesis of GD. Immunochip genetic association analyses have identified 30 single-nucleotide polymorphisms in several genes significantly associated with the young age of onset (AO) GD, i.e. onset <30 years of age, including major histocompatibility complex class I and class II genes, BTNL2, NOTCH4, TNFAIP3 and CXCR4; most of the genes known to be associated with adult-onset GD were also associated with Young AO GD [9]. The epidemiology of GD is the result of complex interactions between genetic, epigenetic and various environmental factors. Gene- gene interactions and gene- environment interactions (e.g. viral infection-related production of interferon-α induced alteration in thyroglobulin gene expression through epigenetic changes in histone modification) are mainly accountable for the pathogenesis. The thyroidal CD40 over expression can augment the severity of GD but is not required for disease development; in mice, it increased the level of thyrotropin (TSH) receptor antibodies and thyroid hormone production [10–14]. In humans, it is strongly associated with persistently high levels of post- treatment thyroid antibodies suggesting a role in thyroid antibody production [15]. The risk of developing GD is greatly increased when two or more disease-associated alleles are inherited together [14].

In the vast majority of cases, GD is the chief cause for thyrotoxicosis in pregnancy. However, as both pregnancy and hyperthyroidism are accompanied by thyroid stimulation, hyperdynamic circulation and hypermetabolism, the detection of hyperthyroidism can be challenging during pregnancy. Biochemically, a serum TSH level lower than the trimester-specific lower limit 0.3 mIU/L and an elevated free T4 level greater than the normal range for pregnancy strongly suggests coexistent hyperthyroidism; the detection of TSH receptor antibodies virtually confirms the diagnosis of GD [7]. In this case, confirmation of GD was achieved by the findings of diffuse toxic goiter in ^99m^ Technetium scintigraphy thyroid scans and highly raised anti thyroid peroxidase antibodies albeit negative TSH receptor antibody.

Severe GD is uncommon in pregnancy as it is related with reduced fertility. For women with milder disease who successfully conceive, hyperthyroidism endows an increased risk of pregnancy loss and established pregnancy complications (Table 1) [7, 16].

**Table 1:** **Potential maternal and fetal complication in uncontrolled hyperthyroidism**

Maternal	Fetal
1. Pregnancy induced hypertension	1. Neonatal hyperthyroidism
2. Pre-eclampsia	2. Intrauterine growth retardation
3. Preterm delivery	3. Small-for-gestational- age
4. Congestive heart failure	4. Prematurity
5. Thyroid storm	5. Stillbirth
6. Increased or recurrent miscarriage	6. Increased perinatal mortality
7. Placenta abruption	
8. Infection	
9. Increased maternal mortality	

In this case, GD was first detected during the third trimester of pregnancy and was complicated by the development of bi- ventricular heart failure, owing to the simultaneous contributions of both thyrotoxicosis and pre-eclampsia. With a blood pressure of 180/100mmHg, we also found a wide pulse pressure in this patient, characteristic of hyperthyroidism. Hyperthyroidism is a secondary cause of isolated systolic hypertension despite low systemic vascular resistance, due to increased arterial stiffness [17, 18]. Cardiac output may be increased by 50 – 300% over that of normal subjects as a result of the combined effect of increase in resting heart rate, left ventricular contractility, ejection fraction and blood volume with a decrease in systemic vascular resistance [19, 20]. Thyroxin not only affects the heart, but also alters the vascular smooth muscle and endothelial cell function via genomic and non-genomic actions targeting membrane ion channels and endothelial nitric oxide synthesis [21, 22]. In addition, research evidence has revealed that the Calcium/ Calmodulin- dependent kinase IV (CaMKIV), which is a major thyroid hormone target gene in the developing brain, plays an important role in blood pressure regulation through the control of endothelial nitric oxide synthase (eNOC) activity. There is also a significant association between the human CaMKIV gene polymorphism and high diastolic blood pressure among hypertensive patients [23, 24]. This patient had high diastolic blood pressure (diastolic blood pressure 100 mm Hg) and there is a possibility of presence of CaMKIV gene polymorphism in this case. Dysfunctional CaMKIV, albeit not expressed in the heart, might partake in cardiac organ damage in the context of the hypertensive state that we found in this case [23].

It has been well established that hyperthyroidism is associated with left ventricular dysfunction and heart failure [25]. β - adrenergic receptors in the myocardium are under thyroid hormone regulation and are positively regulated; the G- protein coupled receptor kinase GRK5 is an important regulator in beta-adrenergic signaling [20, 26, 27].

There is recent emergence of evidence of GD involving the right heart, similar to our case.One study reported that 49% of patients with primary pulmonary hypertension have autoimmune thyroid disease after clinical, biochemical and serologic evaluation [28]. Another study revealed that pulmonary hypertension was detected echocardiographically in 15 patients out of 23 patients (63%) of hyperthyroidism [29]. Albeit unconfirmed, pulmonary hypertension in hyperthyroidism can be postulated by the following: 1) immune mediated endothelial damage or dysfunction; 2) endothelial injury as a result of increased cardiac output; 3) increased metabolism of intrinsic pulmonary vasodilator substance (prostacyclin and nitric oxide) [30–33]. The regression of pulmonary hypertension following maintenance of euthyroid state may support this mechanism.

Concordant with this patient’s finding of MR and TR on Doppler echocardiography, atrioventricular valve regurgitation has been documented to occur in hyperthyroidism with a high prevalence [34–36].

## Conclusions

This was a unique case of GD-related bi- ventricular heart failure, severe pulmonary hypertension and pre- eclampsia in pregnancy. Albeit uncommon, un-explained bi-ventricular heart failure in pregnancy may very well be explained by Graves’ disease. Such a diagnosis requires a high index of suspicion and subsequently specific treatment should be commenced promptly in order to ensure better outcome in such patients.

## Consent

Written informed consent was obtained from the patient for publication of this case report and for all the accompanying images. A copy of the written consent is available for review by the Editor-in-Chief of this journal.

## Abbreviations

CaMKIV: Calcium/ Calmodulin- dependent kinase IV
GRK: G- protein coupled Receptor Kinase
GD: Graves’ disease
MR: Mitral Regurgitation
PASP: Pulmonary Arterial Systolic Pressure
TSH: Thyroid Stimulating Hormone
TR: Tricuspid Regurgitation
Young AO GD: Young age of onset Graves’ disease.
